# Initial romanian experience with green light hps 120 w laser in bph

**Published:** 2008-11-15

**Authors:** Geavlete P., Niţă Gh., Geavlete B.

**Affiliations:** * Department of Urology, „Saint John” Emergency Clinical Hospital, Bucharest, Romania

**Keywords:** potassium-titanyl-phosphate (KTP) laser, photoselective vaporization prostatectomy (PVP), benign prostatic hyperplasia (BPH), 120W Green Light laser

## Abstract

**Introduction and objectives:**Potassium-titanyl-phosphate (KTP) laser photoselective vaporization prostatectomy (PVP) is a relatively new technology for the management of benign prostatic hyperplasia (BPH). We reviewed our initial experience regarding the efficacy and safety of this technique for symptomatic and obstructive BPH.

**Material and methods:** During the last year, 35 patients with a mean age of 65.3 years (between 53 and 80) and symptomatic BPH were treated by laser prostatectomy using KTP/532 laser energy at 120W. The procedure was performed through a 21F continuous-flow cystoscope with normal saline as irrigant. All patients underwent standard urologic evaluation using the International Prostate Symptom Score (IPSS), the urinary peak flow rate (Qmax), ultrasound measurement of prostate volume and residual urine volume, assay of prostate specific antigen (PSA) and digital rectal examination (DRE). The mean prostatic volume was 45cm3 (range 30–70cm3). The patients were reassessed postoperatively at 3 and 6 months.

**Results:** In all cases, KTP laser vaporization was successfully performed, with a mean operating time of 57 minutes (range 20-120 minutes). In most cases, we used just one fibre, the mean energy released being 170.000 Joules (range 80.000-270.000). The mean hospital stay was 24 hours. No major complications occurred intraoperatively or postoperatively, and no transfusions were necessary. All patients were catheter-free after 1 month. At 3 and 6 months, the mean urinary peak flow increased from the preoperative value of 8.5mL/sec to 23.7mL/sec and 21.2mL/sec, respectively. The mean IPSS decreased from 19.0 to 9.5 and 7.5 at 3 and 6 months, and the mean post-voiding residual volume (PVR) decreased from 90.5 to 30.5mL and 15.0 mL. Two patients were admitted for secondary hematuria and urinary infection, and 7 patients presented irritative low urinary tract symptoms during their first postoperative check-up.

**Conclusions:** BPH photoselective vaporization using 120W Green Light laser is a safe and easy to learn technique, with good functional results and a low rate of complications.

## Introduction

The gold standard treatment of benign prostatic hyperplasia (BPH) is represented by the transurethral resection of the prostate (TURP). Alternative therapies have been developed aiming to reduce the level of complications while maintaining efficacy. These methods included microwave therapy, transurethral needle ablation, and a range of laser procedures (laser prostatectomy - LP). LP involves tissue coagulation or vaporization. Tissue coagulation results in gland debulking by necrotic tissue sloughing, while vaporization produces an instantaneous debulking of prostatic tissue.

The latest addition to laser therapy for BPH is photoselective vaporization of the prostate (PVP). Since its introduction in 1998 by Malek et al [**1**], PVP was characterized by a relatively simple technique, excellent clinical outcomes, low morbidity and cost effectiveness. The use of Green Light led to a considerably increased success in the field of LP [**2**]. This procedure results in rapid vaporization of prostatic tissue, with good outcome reported after up to 5 years of follow-up.

In our country, PVP was performed as a national premiere in the Department of Urology of „Saint John” Emergency Clinical Hospital.

## Materials and Methods

During the last year, 35 patients with a mean age of 65.3 years (between 53 and 80) and symptomatic BPH were treated by PVP.

All patients underwent standard urologic evaluation using IPSS, Qmax, ultrasound measurement of prostate volume and residual urine volume, PSA and DRE. The mean prostatic volume was 45cm3 (range 30–70 cm3).

Patients with a Qmax < 15mL/sec or ultrasonographically measured PVR > 70mL in conjunction with an IPSS > 7 were considered to be suitable candidates for laser therapy.

In addition, the use of anticoagulants didn’t represent an exclusion criterion, nor did any history of acute urinary retention. 6 of the treated patients were in retention, 7 patients were receiving anticoagulants, and 5 patients had prostates > 50mL.

Patients with suspected prostate cancer based on elevated PSA values or a suspicious DRE were excluded from treatment.

All procedures were performed under spinal anesthesia with normal saline as irrigant.

**Fig. 1:**
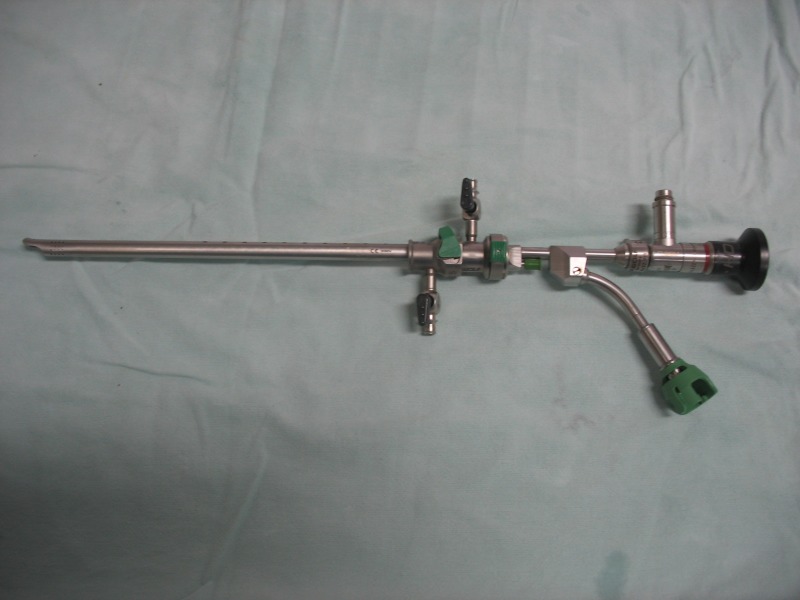
The new Green Laser Wolf cystoscope

The first 20 procedures were performed through a 21F continuous-flow cystoscope. For the last cases, we used the new green laser Wolf cystoscope (22.5F) (**Fig. 1**).

**Fig. 2:**
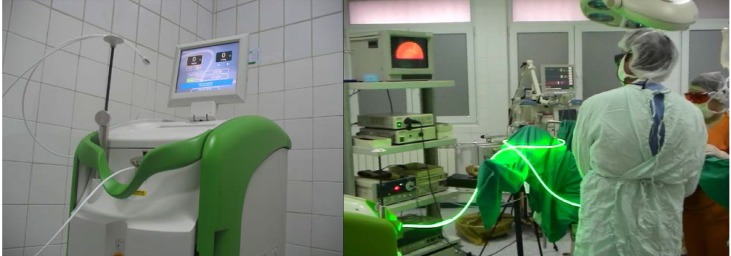
Green Light HPS (High Performance System) 120W Laser

All cases were treated with the new Green Light HPS (High Performance System) 120W Laser (**Fig. 2**). This system operates with a fiber that emits a beam which is more collimated and more powerful than the old 80-W KTP laser.

Generally, the power setting used at the beginning of the procedure was 80W, and was afterwards increased to 100W and finally to 120W.

**Fig. 3:**
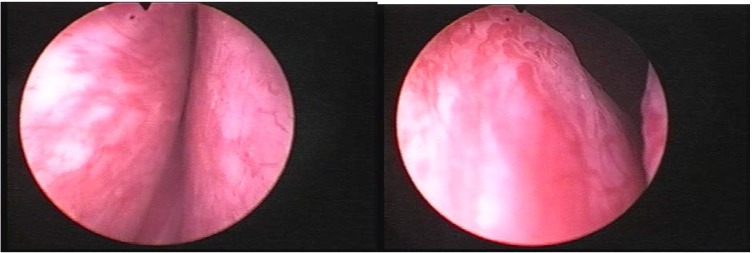
Preliminary cystoscopy, evaluating the prostate size

The first step of the endoscopic procedure was represented by preliminary cystoscopy in order to evaluate the prostate size, length of the prostatic urethra, the presence of a urethral stricture. At the same time, we located the ureteral orifices and the bladder neck (**Fig. 3**). 

The procedure was carefully carried out, thus avoiding any sudden maneuvers which may have determined secondary bleeding, consequently compromising the intraoperative visibility.

The second step included the creation of the working space. Creating a working space when starting the procedure is imperative, as it allows the fiber to move more easily, thus avoiding tissue contact and consequent fiber degradation. This space will provide good orientation during surgery and will also permit optimal irrigation during lasing; an important feature as far as visibility is concerned.

**Fig. 4:**
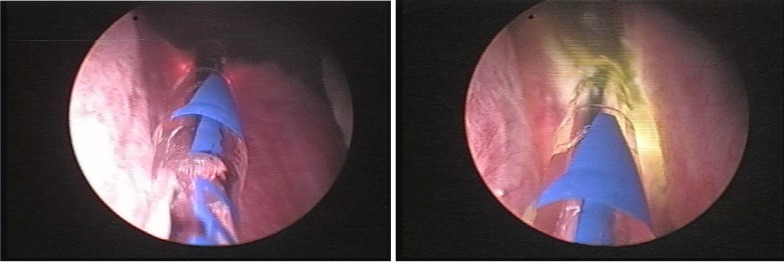
Creating the working space

The working space was generated by central approach (**Fig. 4**). At the end of this operatory stage, we were able to establish the limits for the future vaporization (the bladder neck as superior limit, and the verum as inferior limit). 

**Fig. 5:**
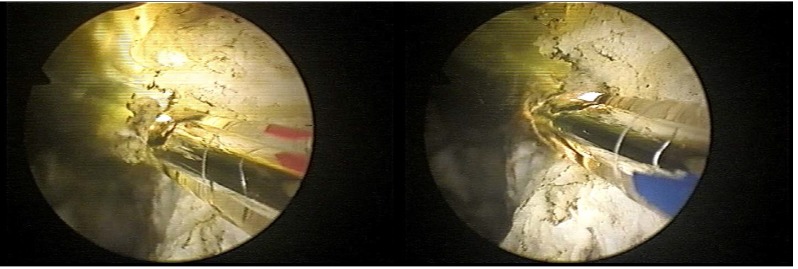
Vaporization of the lateral lobes

The next step was represented by the vaporization of the lateral lobes in a symmetrical manner, layer by layer, aiming to obtain a smooth surface. The objective of this module should be to achieve a concave surface on each side, by removing the lateral prostatic tissue as completely possible (**Fig. 5**). 

**Fig. 6:**
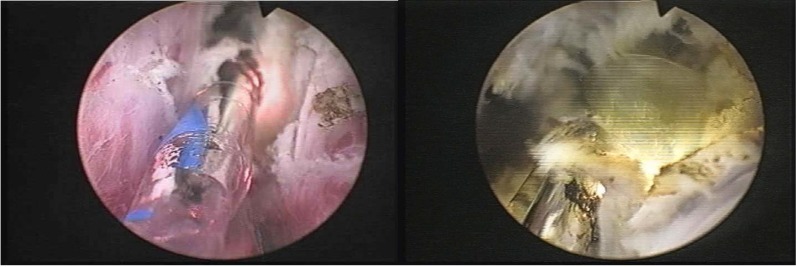
Prostatic apex vaporization

The procedure was finalized by vaporizing the tissue located at the prostatic apex (**Fig. 6**), on each side of the verum, without over passing it, and thus avoiding sphincter lesions.

**Fig. 7:**
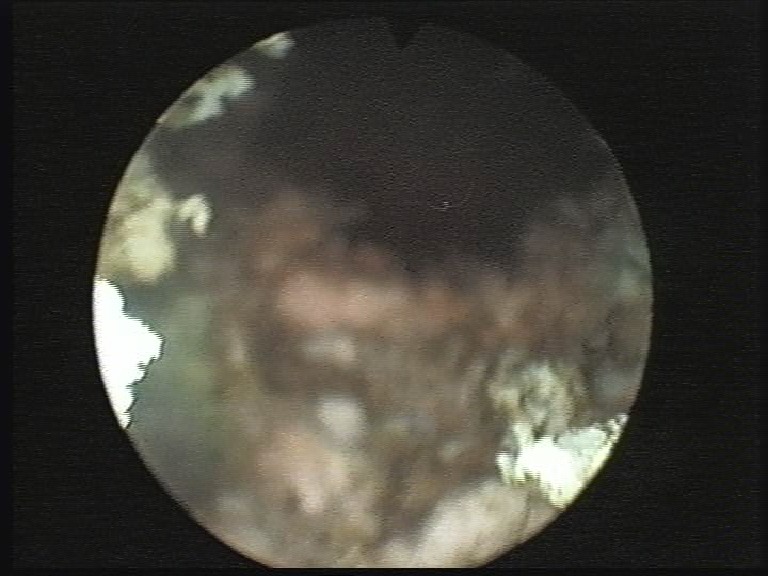
Final postoperative aspect

At the end of the procedure (**Fig. 7**), we completed the hemostasis and mounted a 20 Ch urethral probe.

## Results

In all cases, KTP laser vaporization was successfully performed, with a mean operating time of 57 minutes (range 20 - 120 minutes).

In most cases we used a single fibre, the mean energy released being 170.000 Joules (range 80.000-270.000). 

The mean hospital stay was 24 hours. 

The patients were reassessed postoperatively at 1, 3 and 6 months.

All patients were catheter-free after 1 month. At 3 and 6 months, the mean Qmax increased from the preoperative value of 8.5mL/sec to 23.7mL/sec and 21.2mL/sec, while the mean IPSS decreased from 19.0 to 9.5 and 7.5 at 3 and 6 months. The mean post-voiding residual volume decreased from 90.5 to 30.5mL and 15.0mL, respectively. The results are sumarized in **Table 1**.

**Table 1:** Results after Green Light HPS laser prostatectomy

Parameter	Baseline	Follow-up	
		3 months	6 months
Qmax (ml/s)	8.5	23.7	21.2
IPSS	19.0	9.5	7.5
Post-voiding residual volume (PVR) (mL)	90.5	30.5	15.0

For patients with or without retention, all changes which occurred between baseline and follow-up were significant. With the exception of Qmax, the changes from baseline between groups were not significantly different. 

Patients with or without anticoagulant therapy presented significant improvements over baseline for all measured parameters, and the improvements in all parameters were comparable between the two groups. Patients with a prostate volume > 50 or < 50mL presented significant improvements over baseline for all measured parameters.

No major complications occurred intraoperatively or postoperatively, and no transfusions were necessary. The minor perioperative and postoperative complications are listed in **Table 2**.

**Table 2:** Peri- and postoperative complications associated with the GreenLight HPS 120W laser

Time	Complication	No. Patients
Perioperative	Need for electrocautery to control bleeding	2 (5.7%)
Postoperative	Early dysuria (days 3–14)	7 (17.1%)
	Re-catheterization (retention)	2 (5.7%)
	Urinary tract infection	3 (8.5%)
	Secondary hematuria	2 (5.7%)

## Discussions

The three main types of LP are:

• coagulative with the neodymium:yttrium-aluminium-garnet (Nd:YAG) or the diode lasers;

• cutting (enucleative) with the holmium:YAG (Ho:YAG) or the thulium:YAG (Tm:YAG) lasers;

• vaporisation with the Nd:YAG, Ho:YAG, diode, and the Green Light potassium titanyl- phosphate (KTP) or lithium triborate (LBO) lasers. 

Originally introduced by Malek utilizing a 60W KTP laser, a subsequent pilot study of PVP with the 80W KTP laser showed a good outcome and minimal side effects in men with prostate volumes of 24–76mL [**1**].

PVP works on a different mechanism by comparison to the existing holmium laser enucleation of the prostate, as it involves prostate tissue ablation through vaporization as opposed to enucleation. 

Further progress with PVP was represented by the introduction of the high-performance system (HPS) 120W laser, which aims to reduce lasing time and to improve clinical outcomes while maintaining the same degree of safety for the patients. 

This system generates up to 120W of Green Light (532nm) laser using an LBO crystal, rather than the KTP crystal used in the previous 80W system. It also emits a far more collimated beam through a more efficient laser delivery system.

Concerning the treatment of BPH, the European Association or Urology guidelines state that laser prostatectomy should be advised for patients receiving anticoagulant medication, unfit for TURP (side-fire or ILC), or wanting to maintain ejaculation (side-fire or ILC) [**3**]. The more recent guidelines of the 6th International Consultation on New Developments in Prostate Cancer and Prostate Disease from 2005 state that the KTP laser produced a promising challenge to TURP [**4**].

Since its initial clinical application, numerous studies have been published on PVP, and included long-term outcome at 5 years, comparative studies with regard to TURP, and results in patients with larger prostates, on anticoagulants, as well as in high risk cases.

The functional results of the Green Laser at 3 and 5 years favorably match those of TURP, however describing an essentially improved complications’ profile. Twelve-month outcome was reported by Te (2004) in a multicentre prospective study [**5**]. It emphasized that the mean IPSS score decreased from 23.9 to 4.3, the mean Qmax increased from 7.8 to 22.6mL/sec and the PVR decreased from 114.3 to 24.8mL.

Five-year outcome reported by Malek et al. at Mayo Clinic showed consistent and significant improvements of the key clinical parameters up to 5 years, while using the earlier 60W KTP prototype laser and the later 80W laser [**6**].

Two comparative studies of PVP and TURP showed comparable outcome at 6 [**7**] and 12 months [**8**] with the two therapies. No significant differences were observed between PVP and TURP with regard to IPSS (49.8% versus 50.2% reduction) or Qmax (167% versus 149% increase).

The International Green Light User Group database gathered the results on their initial 305 patients treated with this new higher power laser. Data suggest that a more thorough ablation of the prostate is being carried out with this laser rather than with the original 80W laser [**9**].

A summary of the complications reported with PVP using the 80W KTP laser are shown in **Table 3**.

**Table 3:** Complications associated with Green Laser treatment at 12 months follow-up

	Te (2004) [**10**] No.pts (%)	Sandhu (2004) [**11**] No.pts (%)	Bachmann (2005) [**12**] No.pts (%)	Malek (2005) [**13**] No.pts (%)
Urinary tract infection	4 (2.2%)	1 (2%)	5 (5%)	0
Dysuria	13 (9.4%)	Not available (NA)	6 (6%)	6 (6%)
Incontinence	2 (1.4%)	NA	0	0
Urethral stricture	1 (0.7%)	NA	4 (4%)	0
Bladder neck contracture	2 (1.4%)	3 (3.5%)	1 (0.9%)	2 (2%)
Reoperation	0	3 (5%)	0	0
Recatheterization	7 (5%)	3 (5%)	10 (10%)	1 (1%)

Morbidity is generally low, with reduced rates of incontinence (0–1.4%), urethral stricture (0–4%), and bladder neck contracture (0–3.5%). Re-catheterization and dysuria were more common but consistent with other surgical interventions. 

It is important to underline the fact that the peri- and postoperative transfusion rates were zero in the summarized studies. The procedure is not only successful but also durable, with a reported re-operation rate in the range of 0–5% at 1 year.

A more recent report by Ruszat on PVP use in patients on anticoagulants again confirmed the excellent haemostatic properties of this therapy [**14**]. At 24 months, IPSS improved by 70%, Qmax by 140% and PVR by 80%. The postoperative complications’ rate was low and comparable with the control group. No bleeding complications necessitating blood transfusion or producing clot retention occurred.

With regard to complications, the current 120W laser series appears to be associated with lower rates by comparison to the Ruszat et al. series. The most common complication consisted of transient retention requiring catheterization. Importantly, no blood transfusions were required.

## Conclusions

The specific laser light characteristics and the ideal interactions between KTP lasers and the prostatic tissue result in an even and efficient vaporization of the prostate, as well as in the formation of an obstruction-free prostatic cavity. 

PVP can be a day-care procedure, with few hours of catheterization and minimal postoperative discomfort, offering outcomes at least equivalent to the reference standard TURP. 

However, larger studies are necessary in order to further define the exact place of PVP in the management of BPH. 
